# Supplementary figures and images for: Investigating the impact of motion in the scanner on brain age predictions
Source: Imaging Neurosci (Camb). 2024 Feb 5;2:imag-2-00079. doi: 10.1162/imag_a_00079 (PMC12235560; doi:10.1162/imag_a_00079)

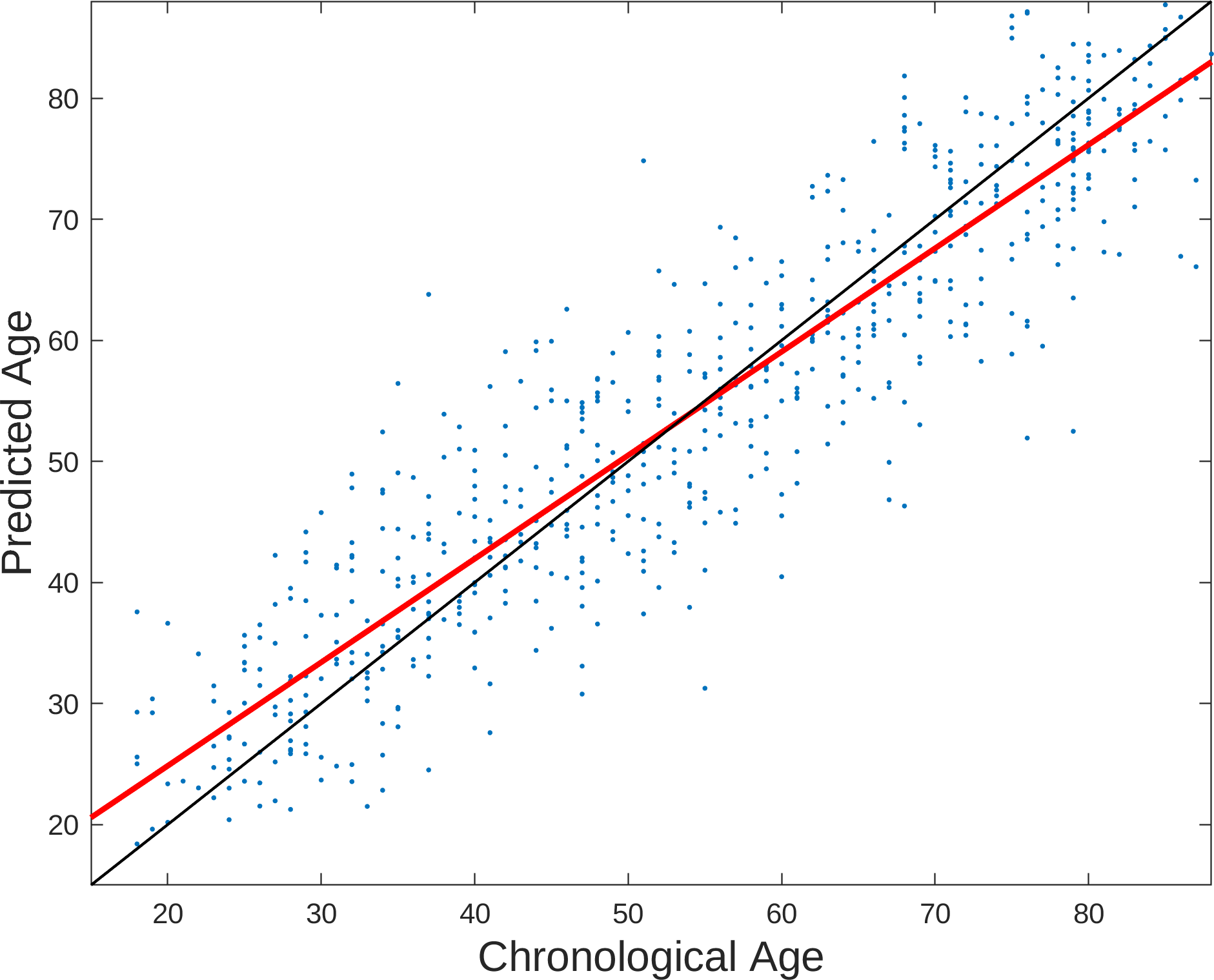

Supplement: Supplementary_Figure_S1 [file imag_a_00079-supp_f1.png]

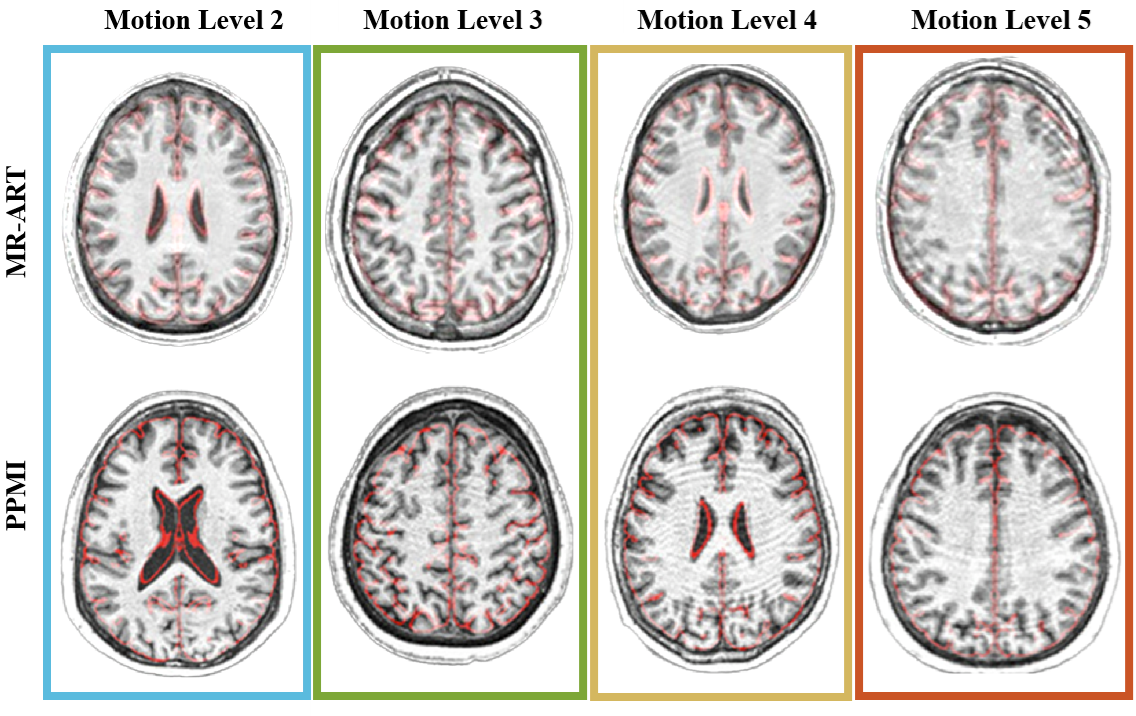

Supplement: Supplementary_Figure_S2 [file imag_a_00079-supp_f2.png]
